# Coastal Land Cover Classification of High-Resolution Remote Sensing Images Using Attention-Driven Context Encoding Network

**DOI:** 10.3390/s20247032

**Published:** 2020-12-08

**Authors:** Jifa Chen, Gang Chen, Lizhe Wang, Bo Fang, Ping Zhou, Mingjie Zhu

**Affiliations:** 1 College of Marine Science and Technology, China University of Geosciences, Wuhan 430074, China; chenjifa@cug.edu.cn (J.C.); fangbo@cug.edu.cn (B.F.); pingzhou@cug.edu.cn (P.Z.); zhumingjie@cug.edu.cn (M.Z.); 2 Key Laboratory of Geological Survey and Evaluation of Ministry of Education, China University of Geosciences, Wuhan 430074, China; lizhe.wang@foxmail.com; 3 School of Computer Science, China University of Geosciences, Wuhan 430074, China

**Keywords:** coastal zone, land cover classification, semantic segmentation, encoder-decoder, context encoding, attention mechanism

## Abstract

Low inter-class variance and complex spatial details exist in ground objects of the coastal zone, which leads to a challenging task for coastal land cover classification (CLCC) from high-resolution remote sensing images. Recently, fully convolutional neural networks have been widely used in CLCC. However, the inherent structure of the convolutional operator limits the receptive field, resulting in capturing the local context. Additionally, complex decoders bring additional information redundancy and computational burden. Therefore, this paper proposes a novel attention-driven context encoding network to solve these problems. Among them, lightweight global feature attention modules are employed to aggregate multi-scale spatial details in the decoding stage. Meanwhile, position and channel attention modules with long-range dependencies are embedded to enhance feature representations of specific categories by capturing the multi-dimensional global context. Additionally, multiple objective functions are introduced to supervise and optimize feature information at specific scales. We apply the proposed method in CLCC tasks of two study areas and compare it with other state-of-the-art approaches. Experimental results indicate that the proposed method achieves the optimal performances in encoding long-range context and recognizing spatial details and obtains the optimum representations in evaluation indexes.

## 1. Introduction

Coastal land cover classification (CLCC) products are indispensable fundamental information in the land–sea junction, which plays a vital role in resource investigation, climate change simulation, and ecological environment protection [1,2,3]. With the remarkable development of data acquisition technologies, satellite, aviation, and other remote sensing platforms have obtained a large amount of high-resolution remote sensing (HRRS) images, providing opportunities for large-scale and high-precision CLCC [4]. However, complicated land cover categories and significant multi-scale features make it difficult to interpret remote sensing images for CLCC. Therefore, using HRRS images to achieve fast and accurate land cover classification is a basic but challenging task.

**Figure 1:**
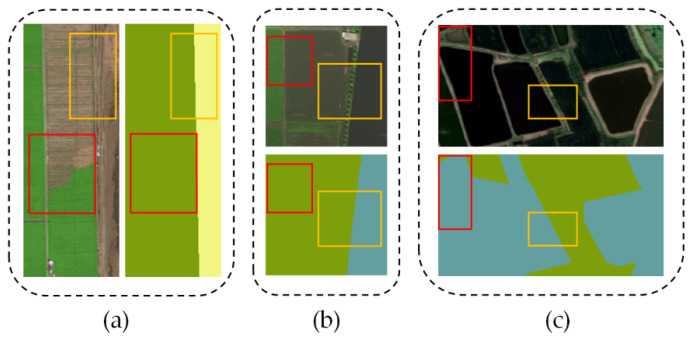
Representative examples of coastal characteristics with low inter-class and high intra-class variance. (**a**–**c**) present different regions and corresponding ground truths, respectively. The yellow box regions represent different land cover categories with similar appearances, while the red box regions represent the same land cover category with different features.

Referring to coastal high-resolution images, there are different spectral, texture, scale, and shape factors for the same category (low inter-class variance). On the other hand, different categories of ground objects display similar feature representations (high intra-class variance) (Figure 1). Notably, it is especially significant for the former. Traditional pixel-based methods distinguish land cover categories by manually extracting feature information such as spectrum, texture, and spatial relationship. These methods mostly include supervised classification algorithms (e.g., KNN, RF, and SVM) and unsupervised methods (e.g., K-means and ISODATA) [5]. However, their feature sensitivity easily leads to incorrect classification, which makes it difficult to meet the requirements of CLCC products with high-precision.

Recently, the fully convolutional neural network (FCN) [6] has made great progress in land cover classification tasks via its powerful capabilities for representing abstract spatial and semantic features. In the implementation, the context reflecting object dependency is an indispensable influencing factor for extracting effective features. Nevertheless, owing to the inherent structure of convolutional operator, FCN-based semantic segmentation models have their receptive field limited to the local region that results in capturing the short-range contextual information. This limitation may cause inconsistencies within classes and affect the accuracy of pixel-level classification. To address this problem, dilated convolution [7] and spatial pyramid pooling [8] have been proposed one after another. The most common strategy is to capture a multi-scale context by fusing dilated convolution or pyramid pooling with different grid ranges [9,10,11,12]. However, dilated convolution is a sparse operation that may cause grid artifacts, since it is unable to extract dense feature information of all positions around the current feature point. Moreover, pyramid pooling may cause a lack of spatial positioning information of pixels [13]. Furthermore, concentrating on the attention mechanism, some works employed recurrent neural networks with long-range dependencies to capture the feature context [14,15], but the effectiveness of these methods depends on the learning results of long-term memorization.

In addition, the ultimate goal of the CLCC task is to obtain the segmentation map with the original size. A commonly exploited workaround [9,10,11] employed high-level feature maps for a simple up-sampling operation to reconstruct the original resolution. This naive strategy may lead to rough classification results, such as fuzzy category edges and a lack of small-scale objects. Another different line [16,17,18] takes more prominence on optimizing fine-grained details and edge information via integrating low-level feature maps, which relies on the encoder–decoder architecture. Specifically, these methods focused on concatenating low-level and high-level features through skip connection at different scales and have become an effective solution. However, the complicated decoding structure may lead to redundant use of feature information and require a significant amount of computing resources.

To solve the above issues, this paper proposes an attention-driven context encoding network (AdCENet) in an end-to-end fashion, which is structured on encoder–decoder architecture. Specifically, at the head and middle of the decoding path, a position-channel attention aggregation module is embedding to capture global contextual information in spatial and channel domains. Alternatively, each decoding block adopts a global feature attention module to introduce multi-scale spatial detail information by extracting the global context of high-level features to weight low-level features. This lightweight structure will not cause too much computational burden. Meanwhile, multiple cross-entropy objective functions are proposed for multi-scale deep supervision to obtain better network performance. The primary contributions of this paper are as follows:

1. Considering the characteristics of coastal ground objects, a novel attention-driven method for CLCC is proposed, which emphasizes the important role of context encoding information in pixel-level classification tasks.
2. In the decoding phase, the position-channel attention aggregation module and global feature attention module are introduced to perform multi-scale and multi-dimensional global context encoding that enables enhancing the classification consistency. As we know, this is a courageous attempt to apply them to explore better performance for CLCC simultaneously.
3. To achieve better classification results, this paper proposes a multi-scale deep supervision strategy and embeds multi-grid operations in the backbone for optimizing the training process.
4. Experiments in two coastal study areas show that compared with other state-of-the-art semantic segmentation models, AdCENet can effectively improve the classification performance and generate high-precision CLCC products.

The rest of this paper is organized as follows: Section 2 illustrates the related work. Section 3 introduces the method proposed in this paper. Section 4 presents the experiments on two datasets and then discussing them in Section 5. Conclusions are summarized in Section 6.

## 2. Related Work

### 2.1. CLCC Implementation for HRRS Images

CLCC is a pixel-level classification task that provides a comprehensive presentation for coastal ground objects such as the location, shape, and spatial relationship. Their effective information provides guidance for coastal zone research. Even though traditional machine learning methods based on a single sample [19,20] have made great contributions to CLCC, it is undeniable that FCN methods that adopt a large number of samples are achieving excellent performance in this field. The majority of these methods are proposed to focus on the multi-scale features and spatial details of ground objects.

Ground objects belonging to the same category may show different scale features, which is a significant characteristic of HRRS images. To solve the adverse effects that are caused by irregular multi-scale features, Shang et al. [21] aggregated a multi-scale context extraction module and an adaptive feature fusion module. Expanding from the lightweight dense network [22], Liu et al. [23] proposed a relation-enhanced multi-scale convolutional network for land cover classification in urban areas. On the basis of the U-net [16] framework, Guo et al. [24] utilized the attention module to improve the accuracy of building extraction by suppressing the background influence of irrelevant feature regions. Moreover, Cao et al. [25] combined a feature extraction network (Resnet), semantic segmentation network (U-net), and integrated conditional random field for post-processing to achieve tree species classification.

To further identify spatial details, some relevant reports suggested adding boundary detection to segmentation models. Relying on Mask R-CNN [26] architecture, Zhang et al. [27] designed a building extraction framework fused with a Sobel edge detection algorithm to solve the problem of object integrity. Liu et al. [28] developed an edge loss enhancement network that employed multiple weighted edge supervisions to retain spatial boundary information and reduce the interference of ambiguous features. Considering the edge information as a priori knowledge, He et al. [29] proposed an edge FCN for land cover classification of remote sensing images.

In general, the majority of the aforementioned works involve complex decoders or additional iteration modules, which is a time-consuming process. In this paper, we extend the encoder–decoder structure to the CLCC task, whose decoders are mainly composed of lightweight global feature attention modules. The architecture design allows the segmentation network to aggregate multi-scale features and spatial details without consuming too much computing resources.

### 2.2. Contextual Information Aggregation

Contextual information that reflects the dependency relation between image objects plays a key role in scene understanding. For example, the spatial distribution relations of ground objects display the position context in remote sensing images. It is feasible to improve the consistency of pixel classification by enlarging the receiving range to capture long-range context. At present, various FCN-based segmentation models have been proposed to obtain long-range context, including aggregating multi-scale contextual information and embedding attention mechanisms.

To expand the receptive field by replacing the inherent structure of the convolutional operator, inserting dilated convolution into the backbone has become a popular method [10,11]. Additionally, global pooling is widely used in computer vision research [30,31,32], which is capable of capturing the global representation of contextual information. For instance, GCN [33] integrated large convolutional kernels and global pooling to enhance local and global context, respectively. Particularly, the multi-scale context has the capability to facilitate segmentation networks for extracting discriminatory features. Ghias et al. [34] introduced a multi-resolution reconstruction model to generate accurate pixel markers by adopting the Laplacian pyramid. For capturing contextual information in different spatial ranges, Zhao et al. [12] established a pyramid pooling module, while Chen et al. [9] proposed an atrous spatial pyramid pooling module.

Given the capacity of attention mechanisms to model long-range dependencies, some segmentation models employed self-similar methods to aggregate long-range context. On one hand, the attention mechanism calculates feature representations per pixel by assigning weights of all positions. To collect information from other positions, PSANet [35] utilized an adaptive learning attention mask and dual-directions information propagation for predicting the current position. OCNet [36] generated object context mapping for per-pixel by calculating the similarity between the current pixel and other pixels. CCNet [37] simplified the position attention module, where the feature of each position was calculated from a certain number of other positions. On the other hand, attention methods were also applied to obtain long-range dependency in the channel domain. SENet [38] adaptively recalibrated the characteristic response of channels by squeezing and exciting the module to explicitly model the context between channels. EncNet [39] employed a channel attention-based context encoding module to encode global semantic features and selectively emphasized feature maps related to categories.

In this work, motivated by the attention mechanism, we introduced the position-channel attention aggregation module to enhance the feature representation of a specific category in the spatial and channel domains. The cascade structures are deployed in the decoding stage, where the high-level features contain rich semantic information related to land cover categories.

## 3. Methodology

### 3.1. Overview

**Figure 2:**
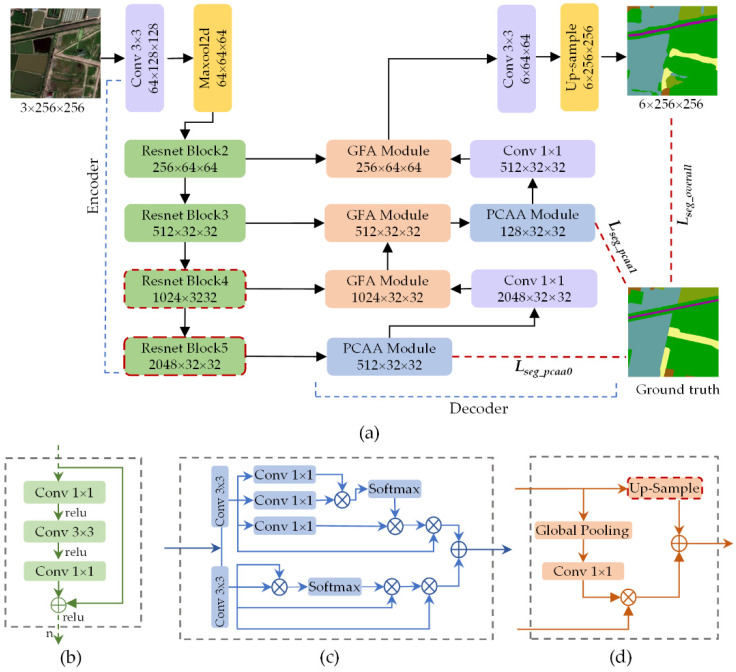
Overview of the proposed attention-driven context encoding network (AdCENet). (**a**) Encoder–decoder paths composed of multiple modules, (**b**) Resnet Block, (**c**) Position–channel attention aggregation (PCAA) module, (**d**) Global feature attention (GFA) module.

Paying attention to the low inter-class variance and complex spatial details of coastal ground objects, a full convolution network based on encoder–decoder architecture and attention mechanisms is proposed for CLCC tasks (Figure 2). On the whole, our proposed AdCENet is primarily comprised of a feature extraction path (encoder) and an up-sampling path (decoder). Furthermore, skip-connections are utilized to map the corresponding low-level features and high-level features in parallel paths, as illustrated in Figure 2a.

In the encoding path, a pre-trained residual network (Resnet101) [31] (Figure 2b) serves as the backbone for feature extraction, following previous works [39,40]. Particularly, concentrating on retaining more spatial detail information without changing the scale of pre-trained parameters, down-sampling layers in the last two residual blocks are replaced with dilated convolutional layers. This strategy makes the size of the output feature map 1/8 of the original image, which can retain more category information.

The decoding path is primarily connected by global feature attention (GFA) modules and position-channel attention aggregation (PCAA) modules in a certain order. Firstly, corresponding to residual blocks of Resnet101, three GFA modules (Figure 2d) are defined to enhance the recognition ability of multi-scale objects and detailed information by generating global feature guidance. Then, focusing on the local receptive field caused by convolutional operators, PCAA modules (Figure 2c) are embedded at the head and middle of the decoding path to capture long-range contextual information in spatial and channel domains, respectively. Additionally, behind each PCAA module, a convolutional layer with a kernel size of 1 × 1 is designed for channel dimensionality transformation to match the low-level feature map. Finally, following the output semantic feature map, a convolutional layer with a kernel size of 3 × 3 and a bilinear up-sampling operation are sequentially cascaded to obtain a pixel-level classification map with the original size. It should be noted that the BN [41] operation and Relu [42] activation function are loaded behind each convolutional and transposed convolutional layer.

### 3.2. Residual Learning Framework

The deep convolution neural network has achieved great breakthroughs in land cover classification tasks, which allows it to learn and capture rich spatial and semantic features. It has been proved that a deeper network can lead to a better ability for feature recognition [43,44]. However, it may cause degradation problems, such as gradient poor training, vanishing gradient, and exploding gradient. Therefore, we employ a pre-trained residual network composed of residual blocks as the feature extraction path to learn effective category features.

**Figure 3:**
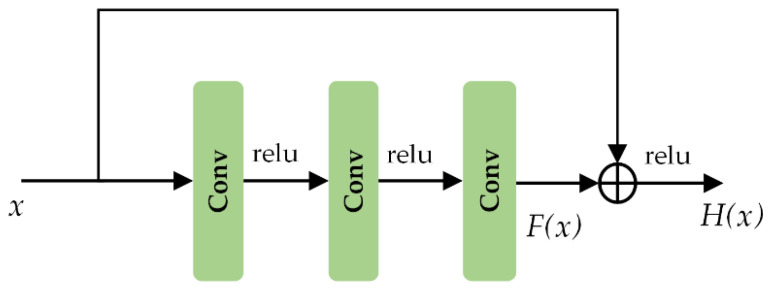
Residual learning: a building block.

As the unit structure of the residual network, the residual block presents the mapping process of several stacked convolutional layers, as illustrated in Figure 3. Denoting the input and mapping output as *x* and *H*(*x*), the *F*(*x*) represents the mapping of stacked nonlinear convolutional operations, formulated as follows:

(1) H(x)=F(x)+x

Notably, even if the convolution layer parameter is 0, it is still an identity mapping. It can be concluded that the residual structure adds identity mapping artificially, and the network performance will not become worse with the increase in depth. On the contrary, the convolutional layers continuously update the weight and iterate in the direction of gradient descent.

### 3.3. Position–Channel Relation Aggregation Module

**Figure 4:**
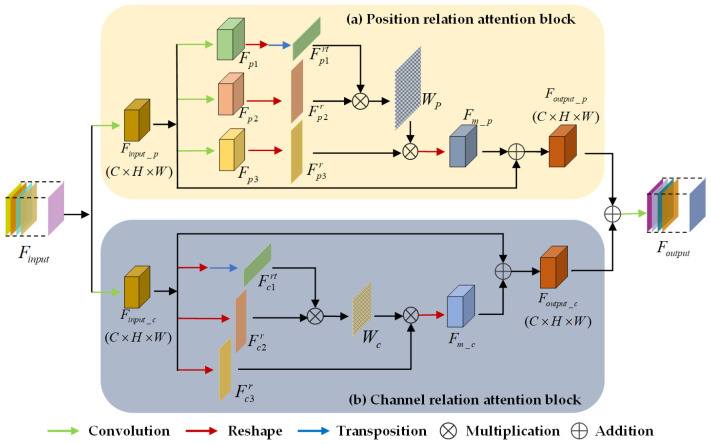
Position–channel attention aggregation (PCAA) module. (**a**) Position relation attention (PRA) block, (**b**) Channel relation attention (CRA) block.

It is an effective way to improve the performance of land cover classification by encoding contextual information to enhance discriminant features. Attention mechanisms enable focusing on the key regions related to a specific category to capture a long-range context. Therefore, a PCAA module similar to proposed in [45,46] recently is introduced in our model, as shown in Figure 4. The module is composed of position relation attention (PRA) block (Figure 4a) and channel relation attention (CRA) block (Figure 4b), which are connected in parallel.

#### 3.3.1. Position Relation Attention Block

The local feature *F_in_p_* *∈* *R^C×H×W^* of channel reduction is given as the input of the PRA block. In addition, three convolutional operators with the same kernel size of 3 × 3 are employed to obtain the feature group *{F_p1_, F_p2_, F_p3_} ∈* *R^C×H×W^*, each with *H × W* spatial dimension and *C* channels. After reshaping them to *{$F_{p1}^{r}$, $F_{p2}^{r}$, $F_{p3}^{r}$} ∈* *R^C×N^* and performing a transposition to $F_{p1}^{r}$, $F_{p1}^{rt}$ *∈* *R^N×C^* is obtained, where *N = H × W*. The spatial dimension attention map *W_p_* *∈* *R^N×N^* allows it to be expressed by a similarity between the current position and other positions in the individual feature map:

(2) $$W_{p}(j,i)=\frac{\exp(F_{p1}^{rt}(i)\cdot F_{p2}^{r}(j))}{\sum_{i=1}^{N}\exp(F_{p1}^{rt}(i)\cdot F_{p2}^{r}(j))}$$

where, $F_{p1}^{rt}$(*i*) and $F_{p2}^{r}$(*j*) denote the elements at the *i*th and *j*th positions in their respective feature maps, and *W_p_* (*j, i*) indicates an impact of the *i*th position on the *j*th position. For the per-pixel of the above attention map, a larger value presents a greater correlation between the features of corresponding positions.

Finally, performing matrix multiplication on $F_{p3}^{r}$ and *W_p_* obtained from the above calculations, and adding their result to the input *F_in_p_*, a position relation feature map *F_out_p_* *∈* *R^C×H×W^* is carried out, as expressed in Equation (3):

(3) $$F_{out\_p}=\lambda_{p}\sum_{i=1}^{N}(W_{p}(j,i)\cdot F_{p3}^{r}(i))+F_{in\_p}$$

where *λ_p_* denotes the learnable scaling factor with an initial value of 0.

Conventional convolutional operator implements a limited receptive field, which easily leads to poor classifications. Referring to the position attention map, the PRA block selectively aggregates long-range spatial context to enhance the relationship between features with similar semantics in different positions. It brings possibilities to achieve semantic consistencies of the same-type ground objects.

#### 3.3.2. Channel Relation Attention Block

Different from the PRA block, the CRA block directly reshapes the fed local feature map *F_in_c_* to generate a matrix group {$F_{c1}^{r}$*, $F_{c2}^{r}$,* $F_{c3}^{r}$} *∈* R*^C×N^* without convolutional operations. After that, matrix multiplication is conducted between $F_{c1}^{rt}$ and $F_{c2}^{r}$, while $F_{c1}^{rt}$ is obtained by transposing $F_{c1}^{r}$. The channel dimension attention map *W_c_* *∈* *R^C×C^* is generated via a softmax function as follows:

(4) $$W_{c}(j,i)=\frac{\exp(F_{c1}^{rt}(i)\cdot F_{c2}^{r}(j))}{\sum_{i=1}^{C}\exp(F_{c1}^{rt}(i)\cdot F_{c2}^{r}(j))}$$

where, $F_{c1}^{rt}$(*i*) and $F_{c2}^{r}$(*j*) denote the elements at the *i*th and *j*th channels, and *W_c_* (*j, i*) indicates an impact of the *i*th channel on the *j*th channel.

Meanwhile, intermediate feature *F_m_c_* *∈* *R^C×H×W^* is obtained by sequentially performing matrix multiplication and reshaping operations on $F_{c3}^{r}$ and *W_c_*. Finally, the channel relation feature map *F_out_c_* *∈* *R^C×H×W^* is formulated via an element-wise summation, as shown in Equation (5):

(5) $$F_{out\_c}=\lambda_{c}\sum_{i=1}^{N}(W_{c}(j,i)\cdot F_{c3}^{r}(i))+F_{in\_c}$$

where *λ_c_* also presents a learnable scaling factor with an initial value of 0.

As we have seen, high-level features have rich semantic information, each channel of whose is regarded as a specific carrier of certain categories. Additionally, semantic information of different categories has a specific relationship in the channel dimension. The CRA block explores interrelationships of different channels by establishing a weighted model of features and enhances the specific semantic representation of features in the channel domain. Consequently, the method improves the ability to distinguish feature channels that represent different categories.

### 3.4. Global Feature Attention Module

To restore the original size of the final predictive segmentation map, [7,9,10,11] employed a naive decoder to perform bilinear up-sample operation crudely, ignoring the spatial details contained in low-level features. Different from these naive decoding modules, a commonly exploited workaround [16,17] leveraged skip-connection to stitch low-level and high-level feature maps, but the operation increases the volume of model parameters. Therefore, to overcome this limitation, a lightweight GFA module is introduced as a fast and effective decoding block.

**Figure 5:**
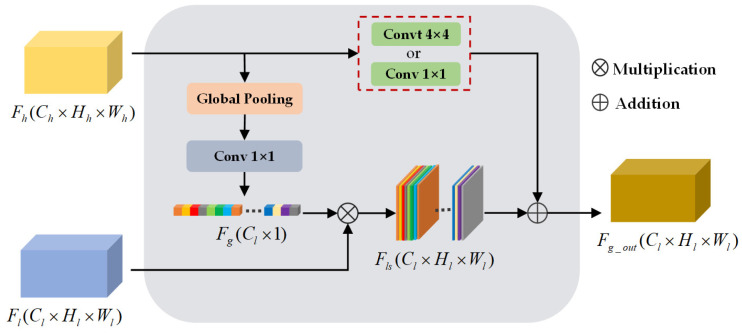
Global feature attention (GFA) module.

As shown in Figure 5, global context *F_g_* *∈ R^C^_l_^×1^* of all channels in the high-level feature map *F_h_∈ R^C^_l_^×H^_l_^×W^_l_* is generated by employing global average pooling:

(6) $$F_{g}(k)=\frac{1}{H\times W}\sum_{i=1}^{H}\sum_{j=1}^{W}F_{h}^{k}(i,j,k)$$

where $F_{h}^{k}$(*i, j, k*) represents the element at position (*i, j*) of the *k*th channel.

Referring to the structural design in this network, the global context *F_g_* and low-level features *F_l_* *∈ R^C^_l_^×H^_l_^×W^_l_* are cascaded to generate a global context-guided spatial detail feature map *F_ls_* *∈ R^C^_l_^×H^_l_^×W^_l_*_._ This process does not require convolution to match the number of channels. Additionally, taking into account the Resnet blocks of the feature extraction path, a transposed convolutional layer is selectively applied to upsample *F_h_* and fuse the corresponding detailed feature map. To achieve this, a semantic feature map *F_g_out_* *∈ R^C^_l_^×H^_l_^×W^_l_* with fine-grained information can be obtained, defined in Equation (7):

(7) $$F_{g\_\mathrm{out}}=\{\begin{array}{cc} convt\left(F_{h}\right)+F_{g}\cdot F_{l}, & upsample=true\\ conv(F_{h})+F_{g}\cdot F_{l}, & others \end{array}$$

where *convt*(⋅) is a transposed convolutional operator with a kernel size of 4 × 4, while *conv*(⋅) presents a convolutional operator with a kernel size of 1 × 1.

In general, the introduced GFA module utilizes the global context provided by high-level semantic features to weight low-level features, which guides the restoration of spatial details such as edge information and small-scale ground objects. It is noteworthy that the module possesses fewer parameters and is able to be selectively deployed in different locations of the decoding stage.

### 3.5. Multi-Scale Supervision

A gradient descent algorithm gradually searches for better parameters by reducing loss objectives. Inspired by recent work [37,46,47], multiple cross-entropy loss functions are leveraged to monitor the features at specific scales in this network. This deep supervision strategy enables the distinguishing of multi-scale features to capture the context of specific categories and optimizing the training process.

The cross-entropy loss function represents a deviation between the predicted value and true label value at each pixel, formulated as follows:

(8) $$L=\frac{1}{N}\sum_{i=1}^{N}\sum_{l=1}^{L}\left(-\left\{y_{l}^{i}\log\left[{\tilde{y}}_{i}\left(l\right)\right]-\left(1-y_{l}^{i}\right)\log\left[1-{\tilde{y}}_{i}\left(l\right)\right]\right\}\right)$$

where *i* and *l* denote the *i*th pixel and *l*th category, and ${\tilde{y}}_{i}$ and $y_{l}^{i}$ indicate the predicted and true value. Our proposed multi-scale supervision establishes a weighted model for segmentation results of the overall network and local two PCCA modules, whose global objective function is expressed as follow:

(9) $$L_{seg\_total}=L_{seg\_overall}+\alpha L_{seg\_pcaa1}+\beta L_{seg\_pcaa2}$$

where *α* and *β* denote the weight coefficients. Referring to the method of specifying parameters in [37], *α* and *β* are set to 0.4 and 0.2, respectively.

## 4. Experiment

### 4.1. Datasets Description

**Figure 6:**
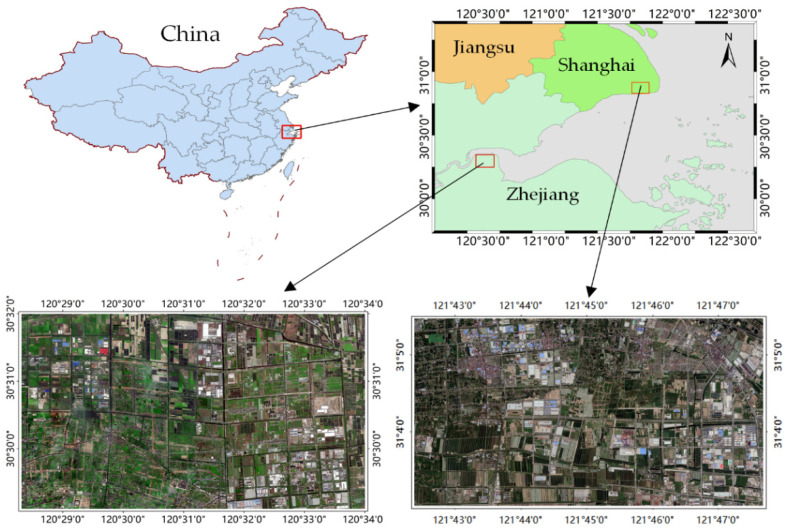
Study areas and corresponding high-resolution remote sensing images.

In this research, two typical coastal areas are selected as the study areas. As illustrated in Figure 6, both the study areas are located on the east coast of China, where moist subtropical monsoon climate results in a variety of ground objects with low inter-class variance and complex detailed information. Specifically, study area I is located in Xiaoshan District, Zhejiang Province. The original data are from aerial images with a high resolution of 0.8 m collected in 2017. The image is composed of three bands of red (R), green (G), and blue (B), covering a scale of approximately 61 square kilometers with a spatial extent of 12,800 × 7424 pixels. Study area II is located in Fengxian District, Shanghai, and the employed satellite images with a resolution of 0.5 m were collected on 26 December 2016. Similar to the image data of study area I, this image contains RGB channels, covering an area of approximately 46 square kilometers with a spatial extent of 18,842 × 9830 pixels. It has been widely accepted that special ground objects in remote sensing images have a constant scale range. Therefore, the image of study area II was resampled to obtain a consistent spatial resolution as study area I. In addition, referring to the above two study areas, there are significant divergences in the spectral, shapes, and scales of all the ground objects. It is primarily caused by the influence of sensors, seasonal factors, and local land-use status. Generally, the selected study areas are characterized by diverse land cover categories and complex spatial distributions, which reflect the unique geographical characteristics of the coastal zone and meet our experimental needs.

**Figure 7:**
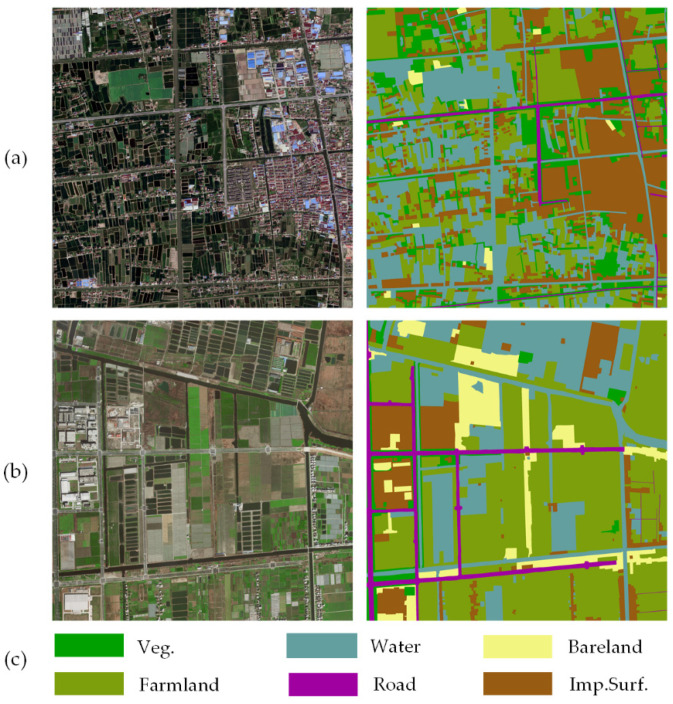
Representative examples of the Shanghai and Zhejiang datasets. (**a**) Aerial image and corresponding ground truth of the Shanghai dataset; (**b**) satellite image and corresponding ground truth of the Zhejiang dataset; (**c**) land cover categories and corresponding annotated color maps.

The Lableme [48] software was used to annotate the image data corresponding to the above two study areas at pixel-level, which is tedious manual work. The generated benchmarks were named Shanghai dataset and Zhejiang dataset, respectively. For both datasets, six land cover categories were defined (Figure 7), including vegetations (Veg.), farmland (Farmland), water (Water), bare land (Bareland), roads (Road), and impervious surfaces (Imp.Surf.). Table 1 gives statistical information on normalized mean, variance, and proportions of all the categories. Notably, it is unbalanced for the land cover categories of both the datasets. For example, the proportions of Road and Bareland are much smaller than Farmland and Imp.Surf. Furthermore, compared with the Zhejiang dataset, the spatial distribution of land cover categories in the Shanghai dataset presents more complex and more detailed information.

### 4.2. Evaluation Metrics

To prove the validity and effectiveness of our proposed AdCENet for CLCC, the multi-categories classification task is taken as multi-segmentation work. Referring to the confusion matrix, TP, TN, FP, and FN denote the numbers of true positives, true negatives, false positives, and false negatives, respectively [49,50]. Thus, the following five metrics are chosen to evaluate the precision of our experimental results.

Per-class Accuracy (PA): Per-class accuracy is the percentage of pixels that are correctly classified in terms of total predicted pixels in each category, as defined in Equation (10):

(10) $$PA=\frac{TP_{c}}{TP_{c}+FP_{c}}$$

where C is the number of land cover categories.

Overall Accuracy (OA): The overall accuracy presents an overall performance of land cover classification models in multi-classification tasks, as defined in Equation (11):

(11) $$OA=\frac{1}{C}\sum_{c=1}^{C}\frac{TP_{c}+TN_{c}}{TP_{c}+TN_{c}+FP_{c}+FN_{c}}$$

Kappa Coefficient (KC): Kappa coefficient is an index for the consistency test. In the classification task, it is used to measure whether the predicted results of the model are consistent with the actual ground truth, as defined in Equation (12):

(12) $$KC=\frac{p_{o}-p_{e}}{1-p_{e}}$$

(13) $$p_{o}=\sum_{c=1}^{C}\frac{TP_{c}+TN_{c}}{TP_{c}+TN_{c}+FP_{c}+FN_{c}},p_{e}=\frac{\sum_{1}^{C}\left(TP_{c}+FP_{c})\cdot (TP_{c}+FN_{c}\right)}{\sum_{1}^{C}{\left(TP_{c}+TN_{c}+FP_{c}+FN_{c}\right)}^{2}}$$

Mean F1 Score (mF1): This metric is regarded as a harmonic average of experimental accuracy and recall rates, as defined in Equation (14):

(14) $$mF_{1}=\frac{1}{C}\sum_{c=1}^{C}2\times \frac{precision\times recall}{precision+recall}$$

(15) $$precision=\frac{TP_{c}}{TP_{c}+FP_{c}},recall=\frac{TP_{c}}{TP_{c}+FN_{c}}$$

Mean IoU (mIoU): IoU is a standard performance measure for object category segmentation, calculating a ratio of the intersection and union of real and predicted categories, while mIoU is a mean value of all the categories, as defined in Equation (16):

(16) $$mIoU=\frac{1}{C}\sum_{c=1}^{C}\frac{TP_{c}}{TP_{c}+FP_{c}+FN_{c}}$$

### 4.3. Experimental Setup

Two comparative experiments were conducted on the aforementioned two datasets to verify the accuracy and generalization of our proposed method. Each original image and the corresponding ground truth of the study areas were clipped into smaller blocks with a size of 256 × 256 by employing a sliding window. The extracted RGB image patches and corresponding ground truth were taken as network inputs, respectively, where the ratio of training set to validation set was approximately 2:1. Specifically, for the Shanghai dataset, the number of the training set and validation set is 752 and 352, respectively, while the corresponding number is 994 and 456 in the Zhejiang dataset. Meanwhile, mean subtraction and normalization were performed on the input images to accelerate the convergence of weight and deviation parameters in the network.

The training set in the two datasets is relatively small, especially the Shanghai dataset. Since data augmentation is an effective method to expand the training set and ultimately improve the robustness, several data augmentation methods were adopted to increase the diversity of our training set in runtimes. These effective methods mainly include random horizontal flip, random vertical flip, and random scaling (from 0.5 to 2.0).

Our proposed method was implemented on the deep learning framework PyTorch [51]. All experiments were performed on a computer with an Intel Core i7-9700k CPU, 16 GB RAM, and NVIDIA RTX 2080 GPU (8 GB memory). Specifically, we set 120 training epochs to achieve an overall convergence of this network with a batch size of four. The stochastic gradient descent (SGD) with a momentum of 0.9 and a weight decay of 0.00001 was used as the optimizer. Additionally, the initial learning rate was set to 0.006 and we employed a “poly” learning rate policy where the learning rate was multiplied by (1-current_epoch/totle_epoch)^0.9^ after each training epoch.

### 4.4. Results and Analysis

In the experiments, several state-of-the-art semantic segmentation models concentrating on multi-scale objects and encoding long-range context were introduced and compared with our proposed AdCENet, including FCN [6] based on Resnet101, RefineNet [18], GCN [33], PSPNet [12], Deeplab V3+ [11], OCNet [36], and EncNet [39]. For all the competitive methods, we utilized pre-trained Resnet101 as the feature extraction network and performed land cover classification tasks on the same datasets.

**Figure 8:**
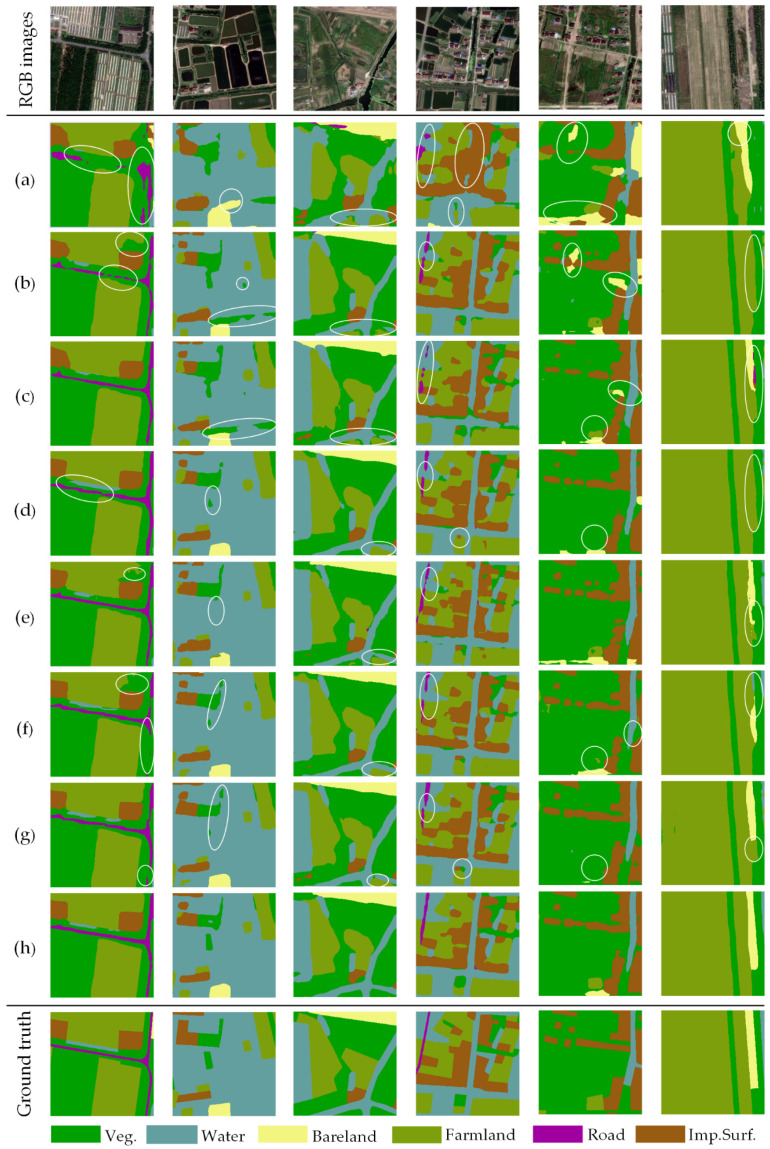
Representative examples of land cover classification results on the Shanghai dataset: (**a**) fully convolutional neural network (FCN), (**b**) RefineNet, (**c**) GCN, (**d**) PSPNet, (**e**) Deeplab V3+, (**f**) OCNet, (**g**) EncNet, (**h**) our AdCENet. The negative results of the aforesaid classification task are circled in white ellipses.

**Figure 9:**
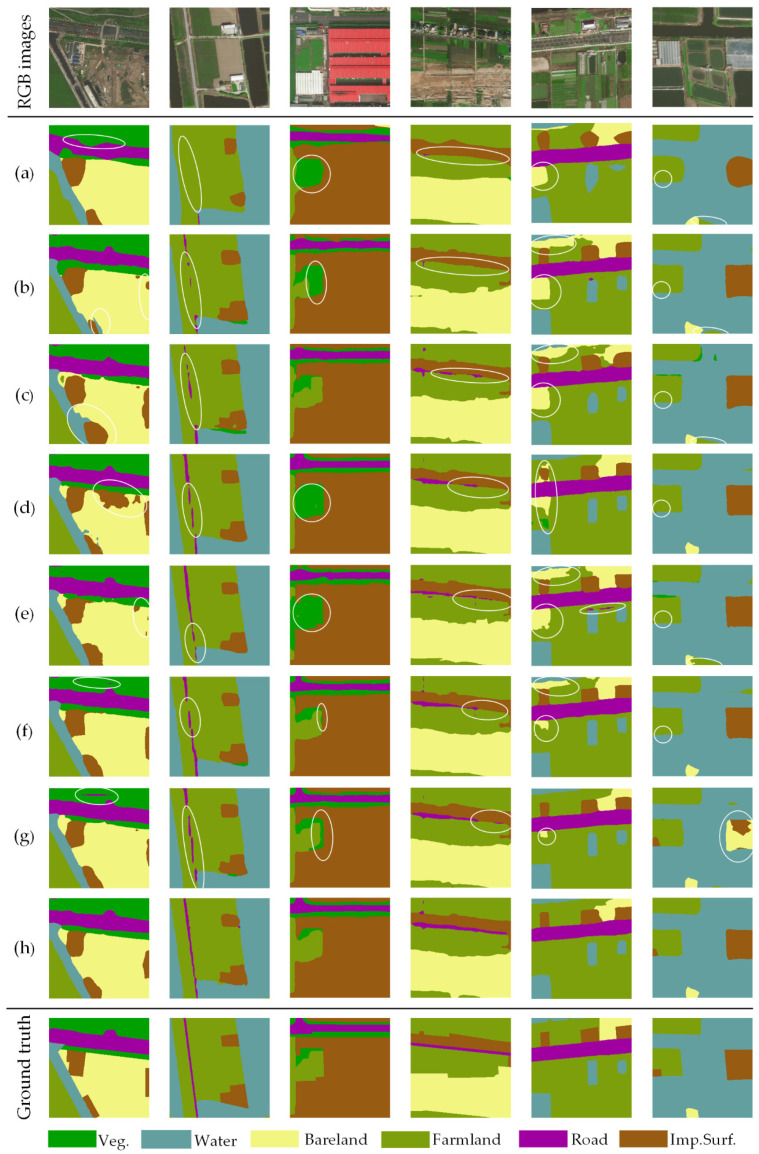
Representative examples of land cover classification results on the Zhejiang dataset: (**a**) FCN, (**b**) RefineNet, (**c**) GCN, (**d**) PSPNet, (**e**) Deeplab V3+, (**f**) OCNet, (**g**) EncNet, (**h**) our AdCENet. The negative results of the aforesaid classification task are circled in white ellipses.

As illustrated in Figure 8 and Figure 9, partial representative examples of CLCC results generated from AdCENet and other competitive methods are expressed. Presented in Figure 8a and Figure 9a, there is no doubt that FCN gains the worst classification effect. Even though FCN is a pioneering work of fully convolutional networks, it ignores the detailed information provided by the decoding path. Compared with FCN, RefineNet and GCN improved the capability to discriminate small-scale ground objects. However, these two methods still have shortcomings in analyzing categories with low inter-class variance (Figure 8b and Figure 9b,c). Moreover, as illustrated in Figure 8d and Figure 9d,e, PSPNet and Deeplab V3+ successfully capture the multi-scale features of ground objects, which utilized dilated convolution and pyramid pooling to overcome the local receptive field of the convolutional operator. However, it is difficult for them to distinguish different ground objects and boundaries with similar features. As the representative works of the attention mechanism, OCNet and EncNet can accurately solve the issue of low inter-class variance, but there are still incorrect classification results (Figure 8f,g and Figure 9f,g). As we expected, the proposed AdCENet achieved an optimal classification result, as shown in Figure 8h and Figure 9h. Specifically, the proposed method outstandingly identifies small-scale features and their boundaries information and achieves excellent performance in recognition of similar but distinct ground objects. It can be concluded that our proposed method comprehensively considers the characteristics of multi-scale and low inter-class variance in HRRS images.

Moreover, Table 2 and Table 3 give evaluation results of all the competitive methods in terms of per-class accuracy (PA), overall accuracy (OA), kappa coefficient (KC), mean F1 Score (mF1), and mean Iou (mIoU) on the Shanghai and Zhejiang dataset. The experimental results show that our proposed AdCENet achieves remarkable performance. For instance, the proposed method acquires the highest OA, KC, mF1, and mIoU values of 93.34%, 91.32%, 92.29%, and 85.81% on the Shanghai dataset and 95.63%, 93.86%, 93.88%, and 88.62% on the Zhejiang dataset. Compared with the well-known Deeplab V3+ [11], AdCENet achieves 3.31% and 1.14% improvement on mIoU, respectively. In terms of PA, AdCENet still obtains the best representations. Specifically, for the categories with larger proportions (e.g., Water, Imp.Surf., and Farmland), the accuracies of all the methods have little divergence, while most of the state-of-the-art models achieve strong recognition ability to the features of large-scale ground objects. However, the proposed network remarkably improved the classification performance on small-scale objects (e.g., Road, Veg., and Bareland), compared with other state-of-the-art methods. It is undeniable that the context encoding method possesses effectiveness in recognizing long-range dependence and spatial details.

**Figure 10:**
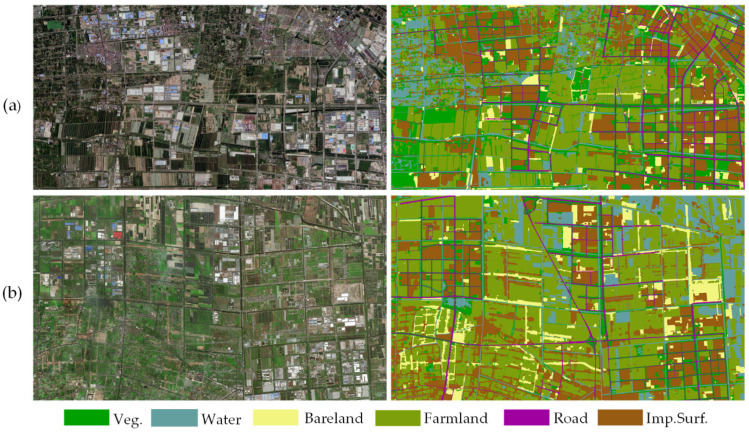
Coastal land cover classification products using our AdCENet. (**a**) Study area I, (**b**) study area II.

Since the prospective aim of our proposed method is to serve the large-area and high-precision CLCC task, Figure 10 gives the land cover classification products of the above study areas. It is worth encouraging that the products provide high-precision spatial distributions of ground objects, which are capable of guiding social and economic activities such as land resource surveys and ecological environment protection.

## 5. Discussion

### 5.1. Ablation Study for Attention Modules

#### 5.1.1. Effectiveness of Attention Modules

At the head and middle of the decoding path, PRA and CRA blocks were embedded to capture the long-range context in spatial and channel domains, respectively. Meanwhile, multi-scale GFA modules were employed as the decoding blocks to fuse low-level detail features. To verify the effectiveness of the aforementioned attention modules, several ablation experiments were carried out on the Shanghai dataset under different settings.

As illustrated in Table 4, attention modules remarkably improve the network performance, while Resnet101 is used as a baseline network. Specifically, the GFA module, respectively, increased by 4.75% and 7.30% in terms of mF1 and mIoU, compared with the baseline. These results strongly prove the superiority of the decoding path constructed by GFA modules. Additionally, the PRA block and CRA block further improved the value of mIoU by 0.86% and 0.56%. As expected, AdCENet, which integrates all the above attention modules, achieves outstanding results up to 81.96% for mIoU.

**Figure 11:**
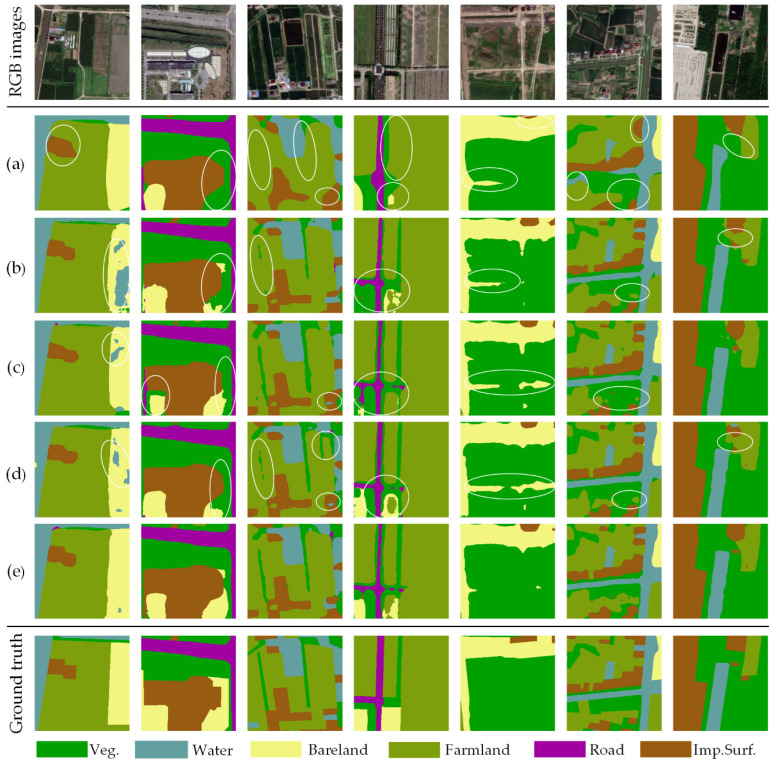
Representative examples of land cover classification results on the Shanghai dataset: (**a**) baseline, (**b**) AdCENet + GFA, (**c**) AdCENet + PRA, (**d**) AdCENet + CRA, (**e**) AdCENet + PRA + CRA. The results of classification errors are circled in white ellipses.

To further analyze the impact of attention modules, several representative examples of land cover classification results are compared, as shown in Figure 11. The baseline without the decoding path gives approximate positions of the land cover categories, and it is difficult to identify small-scale ground objects (Figure 11a). As decoding blocks, GFA modules improve the ability of AdCENet to recognize spatial details, while this method exists poor performance in distinguishing ground objects with low-class variance, as shown in Figure 11b. Furthermore, PRA and CRA blocks improve the representation ability of features with a low inter-class variance to a certain extent (Figure 11c,d), which proves their effectiveness in capturing long-range context. As shown in Figure 11e, AdCENet, integrated by the baseline and all the attention modules, executes a significant improvement in identifying inter-class confusion features and spatial details. In general, each attention module employed in AdCENet enables the network to enhance effective features in different domains or scales and ultimately improve the classification ability.

#### 5.1.2. Influence of Connection Mode

The proposed AdCENet in previous sections is based on an assumption that PRA and CRA blocks in PCAA modules are connected in parallel, which does not consider the impact of different connection modes on network performance. Therefore, a feasibility study was conducted by utilizing several different connection modes on the Shanghai dataset. The connection modes mainly include series connections (Series1: PRA~CRA, Series2: CRA~PRA) and a parallel connection (Parallel: PRA + CRA). Paying attention to the series connections, the PRA and CRA block is arranged in order of presentation.

Table 5 gives the experimental results with evaluation metrics. Compared with Series2, Series1 increases the values of OA and mIoU by 0.22% and 0.46%, which illustrates that it may produce better representation by moving PRA to the head. Meanwhile, the parallel connection employed in our proposed network achieves optimal performance with the highest values of OA, KC, mF1, and mIoU by 91.51%, 88.94%, 89.96%, and 81.96%.

**Figure 12:**
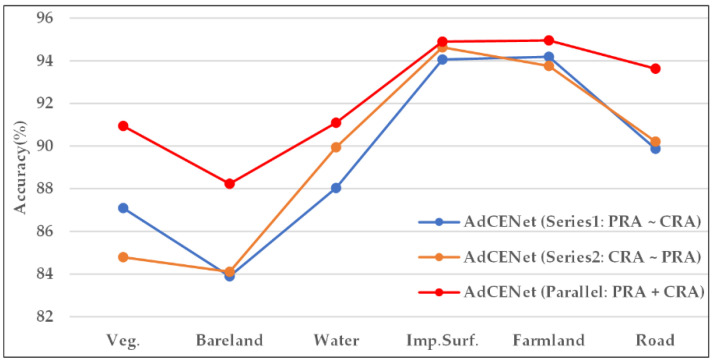
Per-class accuracies of land cover classification on the Shanghai dataset. For all categories, each method with a specific connection mode for the PCAA module corresponds to a broken line of a specific color.

On the other hand, Figure 12 shows the classification accuracy of per-class in a CLCC task by adopting different connection modes. It can be concluded that AdCENet with parallel connection obtains the best accuracies in per-class, while the divergence is especially significant in small proportion and small-scale categories such as Veg., Bareland, and Road. Consequently, to capture long-range contextual information in both spatial and channel domains, we suggest building the PCAA module in parallel. In this way, AdCENet is pretty good at executing excellent classification ability.

### 5.2. Effectiveness Analysis of Improvement Strategies

In our proposed method, several optimization strategies were introduced to optimize the training process and improve the network performance, including multiple Resnet blocks with dilated convolution (DB), multi-grid (MG) structure for the last Resnet block, and multi-scale deep supervision (DS). In combination with different strategies, several ablation experiments were conducted on the Shanghai dataset to verify their effectiveness.

The proposed AdCENet without any optimization strategy is served as the baseline. Table 6 shows that the last two Resnet blocks with dilated convolutions for retaining the resolution bring improvement by 2.59% for mIoU, while the multi-grid structure has further improved the network performance. It is worth noting that our proposed multi-scale deep supervision significantly enhances the classification ability and achieved approximately 1% improvement and the highest value of 85.81% in terms of mIoU, compared with other methods.

**Figure 13:**
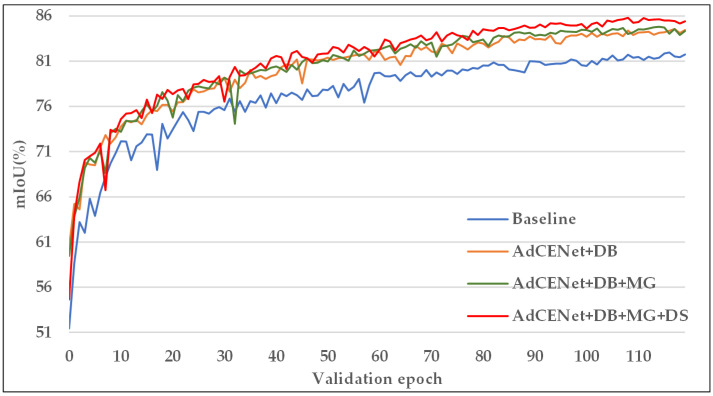
Convergence curve of mIoU for the validation set in each training/validation epoch. Each method with independent improvement strategies corresponds to a curve of a specific color.

Furthermore, Figure 13 shows the convergence curves of mIoU for the validation set under different strategies in each training/validation epoch. In the early epochs of training, the mIoU value of the validation set obtained by employing all optimization strategies is lower than other methods but it will be improved at the fastest speed in the following epochs. After the 40th epoch, our method achieved the remarkable highest value with a smooth boost, while other methods have larger oscillations. Consequently, all the above improvement strategies are effective and practical for our proposed AdCENet.

## 6. Conclusions

This paper proposes a novel AdCENet method for coastal land cover classification from HRRS images. To demonstrate the superiority of the proposed method, another seven state-of-the-art approaches were employed for comparative analysis on the Shanghai and Zhejiang datasets. The experimental results present that the GFA module successfully integrates the spatial details of low-level feature maps by performing global context guidance. Meanwhile, the PCAA module embedded in the decoding stage successfully encodes the global context in the position and channel domains by capturing the corresponding features of a specific category. On the other hand, several ablation experiments were conducted on the Shanghai dataset under different combination settings. The experimental results indicate that the introduced attention modules can effectively improve the classification performance, and the optimization strategies enable it to improve the stability and accuracy of the training process. In summary, the proposed AdCENet achieves better performance in land cover classification with unique coastal characteristics. In the future, we will reduce the model volume to achieve fast and accurate land cover classification under the premise of ensuring network performance.

## Figures and Tables

**Table 1:** Statistics for the Shanghai and Zhejiang datasets.

Statistics	Shanghai Dataset	Zhejiang Dataset
Mean value of each band	0.40, 0.43, 0.32	0.37, 0.37, 0.33
Variance of each band	0.15, 0.13, 0.13	0.19, 0.17, 0.17
Veg.	20.07%	7.86%
Farmland	32.29%	46.03%
Water	11.44%	13.46%
Bareland	3.96%	7.77%
Road	5.79%	4.23%
Imp.Surf.	26.45%	20.65%

**Table 2:** Comparison results to other state-of-the-art methods on the Shanghai dataset (%). The best classification results are highlighted in each column in bold.

Method	Veg.	Bare Land	Water	Imp. Surf.	Farm Land	Road	OA	KC	mF1	mIoU
FCN [6]	77.76	80.73	82.67	91.69	92.70	81.79	87.15	83.23	84.51	73.52
RefineNet [18]	88.07	81.98	88.92	93.42	92.72	91.44	91.00	88.29	89.41	81.11
GCN [33]	87.11	81.43	87.96	93.73	93.62	90.88	91.02	88.29	89.28	80.91
PSPNet [12]	88.07	86.20	91.82	94.85	**95.16**	93.23	92.78	90.59	91.53	84.53
Deeplab [11]	89.71	83.43	90.89	94.15	94.28	91.66	92.34	90.01	91.02	83.70
OCNet [36]	88.31	86.93	91.36	94.51	94.85	92.36	92.57	90.31	91.39	84.28
EncNet [39]	88.51	86.38	**92.14**	94.55	94.84	93.33	92.74	90.54	91.56	84.57
AdCENet	**90.95**	**88.23**	91.10	**94.90**	94.96	**93.63**	**93.34**	**91.32**	**92.29**	**85.81**

**Table 3:** Comparison results to other state-of-the-art methods on the Zhejiang dataset (%). The best classification results are highlighted in each column in bold.

Method	Veg.	Bare Land	Water	Imp. Surf.	Farm Land	Road	OA	KC	mF1	mIoU
FCN [6]	78.30	88.73	89.71	92.52	95.64	79.97	91.64	88.23	87.77	78.75
RefineNet [18]	85.32	89.47	93.01	95.18	96.76	87.70	94.07	91.66	91.60	84.81
GCN [33]	84.02	90.07	93.15	94.64	96.56	89.51	93.90	91.43	91.39	84.46
PSPNet [12]	86.67	91.81	95.28	95.67	97.43	90.62	95.18	93.21	93.21	87.48
Deeplab [11]	87.25	91.72	95.24	95.74	97.23	90.00	95.11	93.12	93.12	87.35
OCNet [36]	84.48	91.71	94.66	95.73	97.42	89.76	94.89	92.81	92.77	86.73
EncNet [39]	85.30	92.90	94.41	95.63	97.43	90.78	95.06	93.04	93.06	87.25
AdCENet	**87.38**	**93.12**	**95.97**	**96.35**	**97.43**	**92.09**	**95.63**	**93.86**	**93.88**	**88.62**

**Table 4:** Performance comparison of AdCENet with different attention modules in evaluation metrics. The optimal evaluation metrics are highlighted in each column in bold.

Method	GFA Module	PRA Block	CRA Block	OA (%)	KC (%)	mF1 (%)	mIoU (%)
Baseline				87.15	83.23	84.51	73.52
AdCENet	√			90.95	88.16	89.23	80.82
AdCENet	√	√		91.33	88.70	89.78	81.68
AdCENet	√		√	91.28	88.63	89.58	81.38
AdCENet	√	√	√	**91.51**	**88.94**	**89.96**	**81.96**

**Table 5:** Performance comparison of attention modules with different connection modes in evaluation metrics. The optimal evaluation metrics are highlighted in each column in bold.

Method	Connection Mode	Order	OA (%)	KC (%)	mF1 (%)	mIoU (%)
AdCENet	Series	PRA~CRA	91.35	88.71	89.80	81.70
AdCENet	Series	CRA~PRA	91.13	88.43	89.50	81.24
AdCENet	Parallel	CRA + PRA	**91.51**	**88.94**	**89.96**	**81.96**

**Table 6:** Performance comparison of AdCENet with different improvement strategies in evaluation metrics. The optimal evaluation metrics are highlighted in each column in bold.

Method	DB	MG	DS	OA (%)	KC (%)	mF1 (%)	mIoU (%)
Baseline				91.51	88.94	89.96	81.96
AdCENet	√			92.80	90.81	91.54	84.55
AdCENet	√	√		93.03	90.92	91.69	84.82
AdCENet	√	√	√	**93.34**	**91.32**	**92.29**	**85.81**
